# Poststroke Depression and Risk of Recurrent Stroke at 1 Year in a Chinese Cohort Study

**DOI:** 10.1371/journal.pone.0046906

**Published:** 2012-10-17

**Authors:** Huai Wu Yuan, Chun Xue Wang, Ning Zhang, Ying Bai, Yu Zhi Shi, Yong Zhou, Yi Long Wang, Tong Zhang, Juan Zhou, Xin Yu, Xin Yu Sun, Zhao Rui Liu, Xing Quan Zhao, Yong Jun Wang

**Affiliations:** 1 Department of Neurology, Beijing Tiantan Hospital, Capital Medical University, Beijing, China; 2 Department of Neurology, Beijing Daxing District Hospital, Capital Medical University, Beijing, China; 3 Institute of Mental Health, Peking University, Beijing, China; University of Münster, Germany

## Abstract

**Background:**

Studies show that poststroke depression (PSD) increases mortality risk at 1 year. However, whether PSD increases the risk of recurrent stroke at 1 year remains unclear. This study was to investigate whether PSD at 2 weeks following a stroke could increase risk of recurrent stroke at 1 year.

**Methods and Results:**

This was a multi-centered prospective cohort study. A total of 2306 patients with acute stroke were enrolled in our study. PSD was diagnosed according to the criteria set by the Diagnostic and Statistical Manual of Mental Disorders, fourth edition (DSM-IV). The outcomes of recurrent stroke were followed up via face-to-face or phone interview. A total of 1713 patients had complete follow-up data, with 481 (28.1%) cases of PSD and 158 (9.2%) cases of cumulative recurrent stroke at 1 year. Multivariate logistic regression analysis showed a 49% increase of OR of recurrent stroke at 1 year in patients with PSD, compared to patients without PSD following a stroke (OR = 1.49, 95%CI: 1.03–2.15). There was no significant correlation between anti-depressant drugs and the risk of recurrent stroke at 1 year following a stroke (OR = 1.96, 95%: CI 0.95–4.04).

**Conclusions:**

Based on the DSM-IV diagnostic criteria, nearly 3 out of 10 hospitalized stroke patients in China were diagnosed with PSD at 2 weeks following a stroke. PSD is associated with a higher risk of recurrent stroke at 1 year. Our study did not find benefit of anti-depressant drugs in reducing such risk.

## Introduction

In China, stroke has surpassed coronary artery disease to be the number one cause of morbidity and mortality of adults [1]. With the advances in diagnostic and therapeutic techniques, the treatment and prevention of stroke have greatly improved, however, recurrent stroke remains a problem requiring immediate attention. A meta-analysis reported that nearly 11.1% of the patients suffered recurrent stroke at 1 year [2]. Previous studies showed an even higher rate of recurrent stroke, reaching 16%, among Chinese stroke patients [3]. Ellekjaer H et al reported a doubled mortality rate in patients with recurrent strokes compared to that of the initial stroke [4].

PSD is the most common psychological disorder among stroke survivors [5]. Studies have showed that PSD increases the mortality rate at 1 year following a stroke [6]. However, whether PSD increases the risk of recurrent stroke requires further investigation. In addition, the conclusions from previous studies on the correlation between depression and recurrent strokes are inconsistent. Some studies have shown that depression increases the risk of first stroke and recurrent stroke by 45% to 80% [7]–[8]. Some other studies failed to show such correlation [9]–[10]. Moreover, although anti-depressant drugs had been historically applied in clinical, there were few studies on the effect of anti-depressant drugs on the recurrent stroke and the conclusions of these reports were not consistent [11]–[12]. This study was to investigate whether PSD at 2 weeks following a stroke increased the risk of recurrent stroke at 1 year.

## Methods

This Prospective Cohort study on the Incidence and Outcome of Patients with Poststroke Depression in China was the first large-scale multi-center prospective cohort study on PSD in mainland China. It was a national, observational, prospective cohort study involving 56 secondary or tertiary hospitals from April 2008 to April 2010. The objective was to analyze the incidence, risk factors, therapeutic status, and effect of PSD on the outcome of stroke. We selected patients with complete follow-up data at each site to analyze and investigate the association between PSD at 2 weeks and the risk of recurrent stroke at 1 year.

### Ethics Statement

Our study obtained the approval from Ethics Committee at each site, in compliance with Declaration of Helsinki. All patients or their legal representatives signed informed consent form (ICFs).

### Subjects

#### Inclusion criteria

(1) Acute stroke, as delineated by the World Health Organization criteria, was defined as rapidly developing clinical symptoms or signs of focal or global disturbance of cerebral function that last more than 24 hours, with no apparent non-vascular causes (primary and metastatic neoplasms, dural hematoma, post-seizure paralysis, head trauma, etc) [13]. Acute stroke included acute ischemic stroke, intracerebral hemorrhage, and subarachnoid hemorrhage; (2) onset of stroke within 14 days; (3) 18 years or older.

#### Exclusion criteria

(1) presence of communicative problems that preclude a psychiatric examination (e.g., due to reduced level of consciousness, severe hearing or visual impairment, aphasia or severe dysarthria, severe cognitive dysfunction); (2) depression or anti-depressant drugs history; (3) illicit drug dependence; (4) loss of follow-up or death.

### Baseline data

Baseline data of patients with acute stroke was collected at admission, including demographics (age, gender), and vascular disease risk factors such as hypertension (history of hypertension or anti-hypertension drug), diabetes (history of diabetes or anti-hypoglycemic drug), hyperlipidemia (history of hyperlipidemia or lipid lowering drug), history of stroke, history of cardiac disease, smoking (smoking at present or within 1 month), and high alcohol consumption (≥5 standard drink/day). Within 24 hours after admission, stroke severity was assessed according to the National Institutes of Health Stroke Scale (NIHSS) [14], and other clinical data, including systolic and diastolic blood pressures at admission and type of stroke was recorded. Baseline data also included intervention measures that patients received, including intravenous thrombolysis, anti-coagulation, anti-platelet, anti-hypertension, lipid lowering drug, anti-hyperglycemic drug, anti-depressant drugs and stroke patient education. Categories and dose of anti-depressant drugs were as follows: Fluoxetine(10–20 mg/d), Paroxetine(10–25 mg/d), Sertraline(25–50 mg/d), Citalopram(10–20 mg/d), Venlafaxine(75–150 mg/d), Mirtazapine(15–30 mg/d). Duration of anti-depressant drugs was determined by the clinicians according to the patients' condition.

All investigators received standard training to proper and consistent application of DSM-IV. Investigators acquired ICFs after the confirmation and registration of eligible subjects, and periodically reviewed medical records of these patients. The data was then recorded on standard clinical study registration forms. A clinical research organization was assigned for data entry, quality control and data analysis.

### Diagnosis of PSD

The neurologists who received standard training assessed patients for the diagnosis of PSD at 2 weeks following stroke onset. PSD was diagnosed by DSM-IV criteria with at least 1 core symptom for at least 2 weeks, 2 or more non-specific symptoms and functional impairment [15]. The core symptoms include depressed mood and loss of interest. The non-specific symptoms included hypophagia or hyperphagia, insomnia or hypersomnia, asthenia, dysthymia or sense of guilt, lack of concentration, death thoughts, retardation or irritability. Other causes such as life stressors (almost exclusively referred to as major or severe negative life events, such as the loss of primary relationship, death of significant others, serious illnesses, major household problems, loss of employment, and so on) were excluded.

### One-year follow-up and assessment of recurrent stroke

The outcome data was obtained at 3 time points (3 months, 6 months, and 12 months) via phone or face-to-face follow-up. The items being assessed were PSD severity degree (Hamilton Rating Scale for Depression (17-items), HRSD-17) [16], modified Ranking Scale (mRS) [17], recurrent stroke, death, etc. Medication should be also recorded. All follow-ups were based upon standard follow-up agreement and performed by clinical physicians who received standard training at each site and were blind to patient's clinical data and whether PSD had been diagnosed.

The recurrent stroke was defined as new neurological symptoms such as sudden aphasia, facioplegia or extremities weakness, etc. or stroke diagnosed at re-admission (ischemic stroke, intracerebral hemorrhage, and subarachnoid hemorrhage) [18]. The recurrent strokes confirmed by face-to-face follow-up were then proved by positive imaging results. The incidence of recurrent stroke at 1 year was a cumulative recurrent stroke rate.

### Statistic analysis

Categorical variables were represented by frequency or percentage, and χ^2^ test was used for inter-block comparison. Mean±standard deviation represented continuous variable, and t-test was used for inter-block comparison. Univariate Logistic regression analysis was used for the assessment of the correlation between PSD and recurrent stroke and the calculation of crude OR and its 95% CI. Multivariates Logistic regression model 1 was adjusted for age and gender, and model 2 was adjusted for age, gender, and risk factors contributing to recurrent strokes (hypertension, hyperlipidemia, diabetes, smoking, high alcohol consumption, history of stroke and cardiac disease) [19], and the variates of significant difference between PSD group and non-PSD group, and the calculation of adjusted OR value and its 95% CI. Bilateral P<0.05 represented significant difference. Our study adopted SPSS17.0 for analysis.

## Results

**Figure 1:**
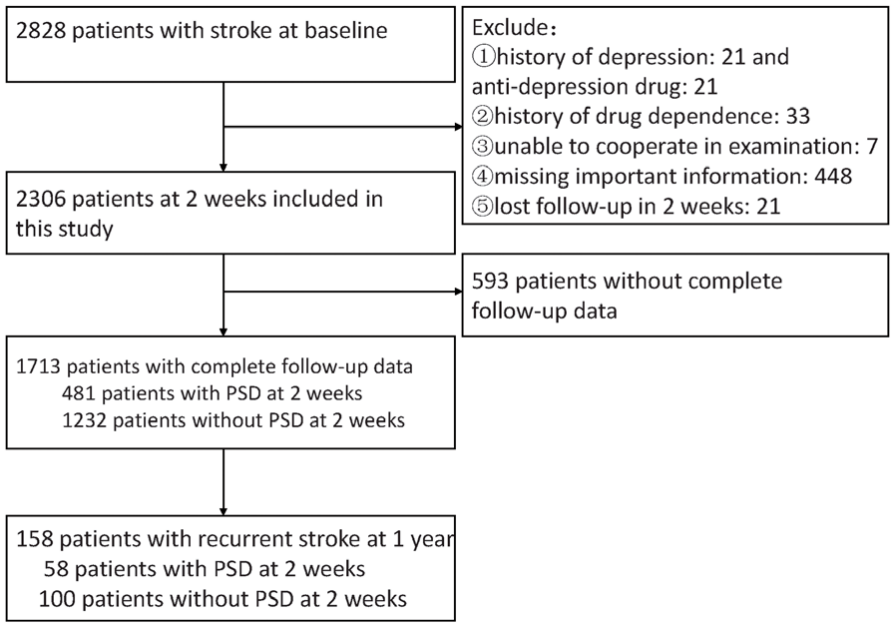
Patients inclusion chart. PSD indicates poststroke depression.

A total of 2828 patients were eligible based on the inclusion criteria, and 2306 patients were enrolled in our study. Patients with history of depression (n = 21), history of anti-depressant drugs (n = 21), and history of drug dependence (n = 33), patients who were unable to finish examination due to severe communicative impairment (n = 7), patients who lost follow-up within 2 weeks (n = 21), and patients who missed important information (n = 448) were excluded (See Figure 1). The percentages of patients with complete follow-up data and patients without complete follow-up data were 1713 (74.2%) and 593 (25.8%) respectively. This article is based on the statistical analysis of patients with complete follow-up data. Among the 1713 patients enrolled, 1708 (99.7%) underwent head CT or MRI to confirm diagnosis. Among those, 502 (29.3%) patients underwent head CT; 1323 (77.2%) patients underwent head MRI; 27 (1.6%) patients underwent CT or MRI but unclear which one; 144 (8.4%) underwent both CT and MRI. 1124(65.6%) were male patients with a mean age of 60.4±11.6 years old and 589 (34.4%) were female patients with a mean age of 63.5±11.3 years old. And there were 1436 (83.8%) cases of ischemic stroke, and 277 (16.2%) cases of hemorrhagic stroke with 247 (14.4%) cases of intracerebral hemorrhage and 30 (1.8%) cases being subarachnoid hemorrhage. 481 (28.1%) patients were diagnosed as PSD at 2 weeks, among those, there were 281(58.4%) male patients aged 60.5±11.4 years old and 200 (41.6%) female patients aged 64.3±11.4 years old. The cumulative recurrent stroke rates at 3 months, 6 months and 1 year were 34 (2.0%), 72 (4.2%) and 158 (9.2%), respectively. There were 27 (1.2%) cases of cumulative deaths at 1 year.

### Difference between patients with and without complete follow-up data

Compared to patients without complete follow-up data, patients with complete follow-up data had lower smoking rate (42.2% vs 49.1%, P = 0.00), anti-depressant drugs (5.8 vs 8.1%, P = 0.00) and stroke patient education rate (84.5% vs 88.5%, P = 0.01), but higher rate for ischemic stroke (83.8% vs 77.7%, P = 0.00) and anti-platelet medication use (81.4% vs 74.7%, P = 0.00). There were no significant difference in age, gender, vascular risk factors including diabetes, hyperlipidemia, hypertension, high alcohol consumption, history of cardiac disease and stroke. In addition, the systolic and diastolic blood pressures at admission, NIHSS at admission, as well as intervention measures including intravenous thrombolysis, anti-coagulation, antihypertensive, lipid lowering drug, and anti-hyperglycemic drug, were not significantly different between the two groups. There was no significant difference in PSD incidence at 2 weeks between the two groups (see table 1).

**Table 1:** Comparison of baseline characteristics between patients with and without complete follow-up data.

Characteristics	Patients with complete follow-up data (n = 1713)	Patients without complete follow-up data (n = 593)	P
Age, y, mean±SD	61.5±11.6	61.4±12.1	0.96
Female (%)	34.4	32.0	0.29
History diabetes (%)	22.9	24.8	0.34
History hyperlipidemia (%)	21.4	19.7	0.39
History hypertension (%)	66.4	65.6	0.73
Smoking (%)	42.2	49.1	0.00*
High Alcohol consumption (%)	3.9	4.2	0.80
History cardiac disease (%)	22.7	21.9	0.71
Personal stroke history (%)	23.2	23.4	0.91
SBP at admission, mmHg, (mean±SD)	151.5±23.9	151.8±23.2	0.79
DBP at admission, mmHg, (mean±SD)	89.1±14.4	88.5±12.8	0.33
NIHSS at admission, median(IQR)	4(2–7)	4(2–8)	0.20
Ischemic stroke (%)	83.8	77.7	0.00*
Intravenous thrombolysis (%)	1.9	2.5	0.37
Anti-coagulation (%)	14.4	16.5	0.21
Anti-hypertension (%)	57.2	54.1	0.20
Anti-platelet (%)	81.4	74.7	0.00*
Lower lipid drug (%)	58.3	56.2	0.37
Lower blood glucose drug (%)	25.0	26.5	0.47
Stroke education (%)	84.5	88.5	0.01*
anti-depressant drugs at 2 weeks(%)	5.8	8.1	0.00*
PSD at 2 weeks(%)	28.1	31.4	0.12

SD indicates standard deviation; IQR, inter-quartile range; NIHSS, national institutes of health stroke scale; PSD, poststroke depression; SBP, Systolic blood pressure; DBP, Diastolic blood pressure.

* indicates significant in statistics.

### Difference of baseline characteristics between patients with and without PSD

Compared to the the non-PSD group, the PSD group had higher percentages of female patients (41.6% vs 31.6%, P = 0.00), higher rate of cardiac disease (26.2% vs 21.3%, P = 0.02) and anti-depressant drugs (13.7% vs 2.7%, P = 0.00), as well as higher initial NIHSS scores (5(2–9) vs 3(1–6), P = 0.00), but lower rates of ischemic strokes (79.2% vs 85.6%, P = 0.00), anti-platelet drug use (77.3% vs 83.0%, P  = 0.00), and lipid lowering drug use(53.2% vs 60.2%, P = 0.00). There were no significant differences between two groups in the following variates: age, hypertension, diabetes, hyperlipidemia, stroke history, smoking, high alcohol consumption, systolic and diastolic blood pressure readings at admission, the use intravenous thrombolysis, anti-coagulation, anti-hypertension, anti-hyperglycemic drug, and stroke education during hospitalization period (see table 2).

**Table 2:** Comparison of baseline characteristics between patients with and without poststroke depression.

Characteristics	PSD(n = 481)	Non-PSD(n = 1232)	P
Age, y, mean±SD	62.1±11.6	61.2±11.6	0.16
Female (%)	41.6	31.6	0.00*
History diabetes (%)	23.1	22.8	0.90
History hyperlipidemia (%)	20.0	21.9	0.37
History hypertension (%)	68.0	65.7	0.37
Smoking (%)	40.7	42.8	0.44
High Alcohol consumption (%)	2.7	4.4	0.10
History cardiac disease (%)	26.2	21.3	0.02*
Personal stroke history (%)	24.1	22.9	0.58
SBP at admission, mmHg, (mean±SD)	151.7±23.9	151.5±24.0	0.87
DBP at admission, mmHg, (mean±SD)	89.1±14.7	89.1±14.3	0.98
NIHSS at admission, median(IQR)	5(2–9)	3(1–6)	0.00*
Ischemic stroke (%)	79.2	85.6	0.00*
Intravenous thrombolysis (%)	1.7	2.0	0.62
Anti-coagulation (%)	15.2	14.1	0.57
Anti-hypertension (%)	57.0	57.2	0.92
Anti-platelet (%)	77.3	83.0	0.00*
Lower lipid drug (%)	53.2	60.2	0.00*
Lower blood glucose drug (%)	24.3	25.2	0.69
Stroke education (%)	85.7	84.1	0.42
anti-depressant drugs at 2 weeks(%)	13.7	2.7	0.00*

SD indicates Standard Deviation; IQR, inter-quartile range; NIHSS, national institutes of health stroke scale; PSD, Poststroke depression; SBP, Systolic blood pressure; DBP, Diastolic blood pressure.

* indicates significant in statistics.

### Correlation between PSD and risk of recurrent stroke within 1 year

Among the 34 patients with cumulative recurrent strokes at 3 months, there were 14 patients (2.9%) with PSD and 20 (1.6%) patients without PSD at 2 weeks. Out of the 72 patients with cumulative recurrent stroke at 6 months, 29 patients (6.0%) were with PSD and 43 (3.4%) patients without PSD at 2 weeks. Regression analysis did not show strong association between PSD and risk of recurrent stroke at 3 months (OR = 1.38, 95%CI: 0.64–2.95), or at 6 months (OR = 1.73, 95%CI: 0.84–3.55).

Among the 158 patients with cumulative recurrent stroke at 1 year, there were 58 cases (12.0%) and 100 cases (8.1%) recurrent stroke in PSD group and non-PSD group respectively. Univariate regression analysis showed that the odds of recurrent stroke at 1 year were 1.55 times greater in patients with PSD than those without PSD (OR = 1.55, 95%CI: 1.10–2.18). In multivariates regression analysis model 1 after being adjusted for age and gender, the OR of recurrent stroke at 1 year of patients with PSD increased by 56% (OR = 1.56, 95%CI: 1.10–2.20) compared to the non-PSD group. In model 2, after being adjusted for age, gender, risk factors for recurrent stroke (such as hypertension, hyperlipidemia, diabetes, smoking, high alcohol consumption, cardiac disease, and stroke history), as well as the variates of significant differences between patients with and without PSD, the OR of recurrent stroke at 1 year of patients with PSD increased by 49% (OR = 1.49, 95%CI: 1.03–2.15) (see Table 3).

**Table 3:** Univariate and multivariates regression analysis for recurrent stroke at 1 year in relation to PSD at 2 weeks.

Model	OR	95%CI
Unadjusted	1.55	1.10–2.18
Adjusted		
Model 1*	1.56	1.10–2.20
Model 2†	1.49	1.03–2.15

PSD indicates poststroke depression.

* : Adjusted by gender and age.

† : Adjusted by gender, age, traditional risk factors for recurrent stroke (including hypertension, diabetes, hyperlipidemia, smoking, high alcohol consumption, personal history stroke, history cardiovascular disease). And other co-variates (including gender, history cardiac disease, NIHSS at admission, ischemic stroke, anti-platelet, lower lipid drug, anti-depressant drugs).

Among 1713 patients with stroke, there were 481 patients with PSD at 2 weeks and 58 cases of cumulative recurrent stroke at 1 year. There were 1232 patients without PSD at 2 weeks, among which, there were 100 cases of cumulative recurrent stroke.

### Correlation between anti-depressant drugs and risk of recurrent stroke at 1 year

There were 481 patients diagnosed with PSD at 2 weeks. Among the 66 (13.7%) patients who received anti-depressant drugs, 12 patients developed recurrent stroke at 1 year. Out of the 415 (86.3%) patients who did not receive anti-depressant drugs, 46 patients developed recurrent strokes at 1 year. Despite anti-hypertension drug treatment was different between patients with and without anti-depressant drugs (74.2% vs 54.2%, P = 0.00), the two groups had no significant differences in other variates (see Table 4). Multivariates regression analysis showed that there was no significant correlation between anti-depressant drugs and the risk of recurrent stroke at 1 year (OR = 1.96, 95%: CI 0.95–4.04) (see Table 5).

**Table 4:** Comparison of characteristics between PSD patients with and without anti-depressant drugs.

Characteristics	Anti-depressant drugs (n = 66)	No anti-depressant drugs (n = 415)	P
Age, y, mean±SD	63.0±11.4	61.9±11.6	0.47
Female (%)	48.5	40.5	0.22
History diabetes (%)	25.8	22.7	0.57
History hyperlipidemia (%)	18.2	20.2	0.69
History hypertension (%)	72.7	67.2	0.37
Smoking (%)	33.3	41.9	0.18
High Alcohol consumption (%)	2.7	4.4	0.10
History cardiac disease (%)	24.2	26.5	0.69
Personal stroke history (%)	22.7	24.3	0.77
SBP at admission, mmHg, (mean±SD)	154.6±26.2	151.2±23.5	0.29
DBP at admission, mmHg, (mean±SD)	88.8±12.3	89.2±15.0	0.84
NIHSS at admission, median(IQR)	5(2–8)	4(2–9)	0.74
Ischemic stroke (%)	80.3	79.0	0.81
Intravenous thrombolysis (%)	1.5	1.7	0.91
Anti-coagulation (%)	19.7	14.5	0.27
Anti-hypertension (%)	74.2	54.2	0.00*
Anti-platelet (%)	78.8	77.1	0.76
Lower lipid drug (%)	59.1	52.3	0.30
Lower blood glucose drug (%)	25.8	24.1	0.77
Stroke education (%)	89.4	85.1	0.35

SD, Standard Deviation; IQR, inter-quartile range; NIHSS, national institutes of health stroke scale; PSD, Poststroke depression; SBP, Systolic blood pressure; DBP, Diastolic blood pressure.

* indicates significant in statistics.

**Table 5:** Univariate and multivariates regression analysis for recurrent stroke at 1 year in relation to anti-depressant drugs in patients with PSD.

Model	OR	95%CI
Unadjusted	1.78	0.88–3.57
Adjusted		
Model 1*	1.81	0.89–3.68
Model 2†	1.96	0.95–4.04

PSD indicates poststroke depression.

* : Adjusted by gender and age.

† : Adjusted by age, gender, traditional risk factors for recurrent stroke (including hypertension, diabetes, hyperlipidemia, smoking, high alcohol consumption, personal history stroke, history cardiovascular disease). And other covariates (including gender, history cardiac disease, NIHSS at admission, ischemic stroke, anti-platelet, lower lipid drug, anti-depressant drugs).

There were 481 cases of PSD at 2 weeks and 66 cases received anti-depressant drugs, among which there were 12 cases of cumulative recurrent stroke at 1 year. Among 415 cases who didn't receive anti-depressant drugs, there were 46 cases of cumulative recurrent stroke at 1 year.

## Discussion

### Basic Characteristics of PSD

Due to variations in diagnostic methods and follow-up durations, PSD incidences in different studies were not consistent. A systematic review showed a PSD incidence of 33% (95%CI 29%–36%) [5]. Our study showed a PSD incidence of 28.1% at 2 weeks after a stroke based on DSM-IV criteria. The PSD incidence in our study was lower, which might because of patients in our study had relatively less severe neurological deficit with a median NIHSS of 4 points. A proportion of these patients with severe neurological impairment were excluded, hence skewing the PSD morbidity rate of general population.

We found, compared to the patients without PSD, PSD was more likely to occur in female patients, patients with hemorrhagic stroke, and patients with higher NIHSS scores. A systematic review showed that female gender, stroke severity and physical disability were all independent predictors of PSD, which were consistent with our study results [20]. In addition, we found that hemorrhagic stroke was a PSD correlative factor too, which might be because hemorrhagic strokes tend to cause more severe neurological impairment than ischemic strokes. The anti-platelet drug and lipid lowering drug use of the patients in PSD group were lower than that of the non-PSD group, which might reflect that the ratio of patients with ischemic stroke were lower in the PSD group than the non-PSD group. There were no significant differences in common risk factors of recurrent stroke, such as hypertension, diabetes, hyperlipidemia, smoking, high alcohol consumption, etc, reflecting that these variates were not closely related to PSD [20].

### PSD and risk of recurrent stroke at 1 year

We found that the odds of recurrent stroke at 1 year were 1.49 times greater in patients with PSD diagnosed at 2 weeks than those without PSD. Mitchell SV Elkind et al. reported that the correlative factors of recurrent strokes included hypertension, hyperlipidemia, diabetes, history of stroke, cardiac diseases including atrial fibrillation and myocardial infarction, smoking and high alcohol consumption, etc [19]. Above mentioned variates were all adjusted in multivariates regression model in our study, the same conclusion was derived in our study, indicating PSD at 2 weeks following a stroke was likely an independent predictor for recurrent stroke at 1 year. Although there were no obvious differences in critical risk factors of recurrent stroke including hypertension, hyperlipidemia, diabetes, history of stroke and cardiac diseases between patients with and without complete follow-up data, since 25.8% of the patients lost follow up, the conclusion derived from our study can not be simply applied to the general population. A more comprehensive follow up is needed to come to a more reliable conclusion.

As far as we know, large scale multi-centered studies investigating PSD and the outcome of recurrent stroke are scarce. In a prospective study by Ruth Peters, et al [8], 2656 elderly patients with hypertension (8.4% had history of stroke) were registered. The Geriatric Depression Score (GDS)≥6 was used to diagnose depression. The average follow-up was 2.1 years. Their multivariates regression analysis showed that depression was related to stroke (HR 1.8, 95% CI 1.2–2.8). Although follow up endpoint of that study combined both first stroke and recurrent stroke, and its diagnostic criteria and follow up duration were different from ours, both conclusions were in parallel. In a prospective study by Mary A, et al [10], 1017 out-patients with coronary disease (14.5% had history of stroke) were enrolled. Patient Health Questionnaire (PHQ) ≥10 was used to diagnose depression. The average follow-up was 4.8 years. They found that the incidence of stroke and transient cerebral ischemia of patients with depression was significantly higher than patients without depression (1.7% vs 0.8%). Their multivariates regression analysis didn't show a strong association between depression and stroke (OR = 1.31, 95%CI: 0.98–1.75). This study showed that OR of the first stroke or recurrent stroke in patients with depression increased 31%, which had no statistical significance. Its conclusion was different from ours, possibly because 100% of the patients enrolled had history of cardiac diseases. Among the subjects in our study, only 22.7% patients with history of cardiac diseases. Cardiac diseases including coronary disease is one of the most important risk factors for recurrent particularly secondary to cardioembolism. In addition, diagnostic criteria, follow up duration, variates in multivariates regression analysis in this study were all different from that of our study, so that it's difficult to directly compare the two studies. Maree et al. pointed out that, in addition to gender, dysfunction severity, cognition impairment, some other risk factors affecting depression also included education, marital status, residence, social status, financial situation and social support, and so on [20]. Therefore, although our study found PSD increased risk of recurrent stroke at 1 year, due to above mentioned uncontrollable and unmeasurable complicating factors, a correlative rather than causative relation exits between PSD and recurrent stroke.

### Anti-depressant drugs and risk of recurrent stroke at 1 year

Three in ten stroke survivors develop PSD in our study, and we found only 14% of patients took anti-depressant drugs, which seemed insufficient in treating PSD in clinical practice. In our study, there was an increase in recurrent stroke in patients with PSD after anti-depressant drugs (OR = 1.96, 95%CI, 0.95–4.04), which had no statistical difference. The span between CI was too wide to make a definite conclusion. Conclusions by previous literature on the effect of anti-depressant drugs on recurrent strokes were inconsistent. Andersen G et al found that there was an increase in recurrent stroke by anti-depressant drugs (OR = 3.09, 95%CI: 0.12–78.70) [11], whereas Lipsey JR et al reported a reduction in recurrent strokes by anti-depressant drugs (OR = 0.41, 95%CI: 0.02–10.69) [12]. However both lacked statistical significance so that it would be difficult to draw a definitive conclusion. A Cochrane review reported that anti-depressant drugs contributed to the remission of PSD symptom and improvement of Hamilton Depression Scale [21]. Our study found that PSD increased risk of recurrent stroke at 1 year, which provided some evidence to speculate that anti-depressant drugs might reduce the risk of recurrent stroke. However, our study results indicated that anti-depressant drugs did not significantly reduce the rate of recurrent stroke at 1 year, which might be related to the following reasons. Firstly, the patients who received anti-depressant drugs were older (mean age 63.0 years old), and there were more vascular risk factors (more than 3/4 with hypertension, and nearly 1/3 with diabetes history and hyperlipidemia history, etc), which significantly independently increased the risk of recurrent stroke. Secondly, among the 66 patients with PSD who received anti-depressant drugs in our study, only 12 patients developed recurrent stroke at 1 year. The small sample size reduced statistical effectiveness. Thirdly, in our study, the anti-depressant drugs were not standardized and only 4.5% of the patients were treated with durations longer than 3 weeks. Nonstandard short-term treatment made it difficult to see long term efficacy. Fourthly, although the mean systolic pressures of the two groups at admission were similar, the rate of anti-hypertension drug treatment in acute phase among patients treated with anti-depressant drugs were much higher than that of patients not treated with anti-depressant drugs. Studies show that excessive reduction of blood pressures during acute stroke stage might exacerbate neurological deterioration [19]. Lastly, anti-depressant drugs have potential side effects that might influence the results [22]–[23]. The correlation between anti-depressant drugs and recurrent stroke requires further clarification by high quality randomized controlled triasl.

### Limitations

This is the first national, multi-centered, prospective cohort study on PSD in China. It is very important for us to explore the affect of PSD on stroke prognosis. Nevertheless, there were limitations in the study that need to be taken into consideration for their influence on the conclusions. Firstly, all patients were extracted from urban hospitals above secondary level. The majority of these hospitals were teaching hospitals, which had more resources and medical professionals compared to the primary hospitals or hospitals in rural areas. Thus, the derived conclusion could only represent the status of hospitals at this level. Secondly, patients with communicative problems, including severe hearing or visual impairment, aphasia or severe dysarthria, and severe cognitive dysfunction, precluding them from a psychiatric examination were excluded from the study. This might underestimate PSD incidence and severity of depression in the general population. Thirdly, asymptomatic silent strokes established by imaging only were not included in the study, which might underestimate the actual rate of recurrent stroke. Fourthly, 39.9% of the patients had unclear recurrent stroke types and 29.7% of the patients had unclear date of recurrent stroke, which made it difficult to make further survival analysis and study the impact of anti-depressant drugs on various recurrent stroke types. Fifthly, due to inherited limitations of non-random observational study, internal validity of our study was limited. Sixthly, the conclusion couldn't be simply extrapolated to all study populations because 25.8% patients were with incomplete follow up information. Lastly, because the study objective didn't include drug dependence, the influence of drug dependence on outcome in our study was unclear. Despite the above mentioned limitations, the investigation into PSD diagnosis at 2 weeks increased risk of recurrent stroke at 1 year, and its various contributing factors are of important value for the clinicians.

### Conclusions

Based on the diagnostic criteria set by DSM-4, nearly 3 out of 10 hospitalized patients with acute stroke in China developed PSD at 2 weeks following the stroke. PSD is associated with a higher risk of recurrent stroke at 1 year. Our study did not find protective effect of anti-depressant drugs on the risk of recurrent stroke at 1 year.
